# Requirement of Histone Deacetylase 6 for Interleukin-6 Induced Epithelial-Mesenchymal Transition, Proliferation, and Migration of Peritoneal Mesothelial Cells

**DOI:** 10.3389/fphar.2021.722638

**Published:** 2021-08-30

**Authors:** Yingfeng Shi, Min Tao, Jun Ni, Lunxian Tang, Feng Liu, Hui Chen, Xiaoyan Ma, Yan Hu, Xun Zhou, Andong Qiu, Shougang Zhuang, Na Liu

**Affiliations:** ^1^ Department of Nephrology, Shanghai East Hospital, Tongji University School of Medicine, Shanghai, China; ^2^ Department of Immunology and Microbiology, Shanghai Institute of Immunology, Shanghai Jiao Tong University School of Medicine, Shanghai, China; ^3^ Emergency Department of Critical Care Medicine, Shanghai East Hospital, Tongji University School of Medicine, Shanghai, China; ^4^ School of Life Science and Technology, Advanced Institute of Translational Medicine, Tongji University, Shanghai, China; ^5^ Department of Medicine, Rhode Island Hospital and Alpert Medical School, Brown University, Providence, RI, United States

**Keywords:** histone deacetylase 6, interleukin-6, epithelial-mesenchymal transition, proliferation, migration, peritoneal mesothelial cells

## Abstract

**Aims:** Influenced by microenvironment, human peritoneal mesothelial cells (HPMCs) acquired fibrotic phenotype, which was identified as the protagonist for peritoneal fibrosis. In this study, we examined the role of histone deacetylase 6 (HDAC6) for interleukin-6 (IL-6) induced epithelial-mesenchymal transition (EMT), proliferation, and migration of HPMCs.

**Methods:** The role of HDAC6 in IL-6-elicited EMT of HPMCs was tested by morphological observation of light microscope, immunoblotting, and immune-fluorescence assay; and the function of HDAC6 in proliferation and migration of HPMCs was examined by CCK-8 assay, wound healing experiment, and immunoblotting.

**Results:** IL-6 stimulation significantly increased the expression of HDAC6. Treatment with tubastatin A (TA), a highly selective HDAC6 inhibitor, or silencing of HDAC6 with siRNA decreased the expression of HDAC6. Moreover, TA or HDAC6 siRNA suppressed IL-6-induced EMT, as evidenced by decreased expressions of α-SMA, Fibronectin, and collagen I and the preserved expression of E-cadherin in cultured HPMCs. Mechanistically, HDAC6 inhibition suppressed the expression of transforming growth factor β (TGFβ) receptor I (TGFβRI), phosphorylation of Smad3, secretion of connective tissue growth factor (CTGF), and transcription factor Snail. On the other hand, the pharmacological inhibition or genetic target of HDAC6 suppressed HPMCs proliferation, as evidenced by the decreased optical density of CCK-8 and the expressions of PCNA and Cyclin E. The migratory rate of HPMCs also decreased. Mechanistically, HDAC6 inhibition blocked the activation of JAK2 and STAT3.

**Conclusion:** Our study illustrated that IL-6-induced HDAC6 not only regulated IL-6 itself downstream JAK2/STAT3 signaling but also co-activated the TGF-β/Smad3 signaling, leading to the change of the phenotype and mobility of HPMCs. HDAC6 could be a potential therapeutic target for the prevention and treatment of peritoneal fibrosis.

## Introduction

Peritoneal dialysis (PD) is an effective and home-based renal replacement therapy for end-stage renal disease (ESRD) patients (Li et al., 2017). Peritoneal fibrosis (PF) is the main factor for ultrafiltration loss and treatment failure in PD patients, and it is characterized by the loss of mesothelial cells (MCs) and increase of myofibroblasts in submesothelial areas, where epithelial-mesenchymal transition (EMT) occurs (Zhou Q. et al., 2016).

The EMT process refers to the *trans*-differentiation of epithelial cells into motile mesenchymal cells, which is regulated by related transcriptional factors, such as Snail and Twist, resulting in upregulation of mesothelial cell markers (i.e., α-SMA, Collagen I, and Fibronectin) and downregulation of epithelial cell markers (i.e., E-cadherin and ZO-1) (Lamouille et al., 2014). Meanwhile, peritoneal mesothelial cells under phenotypic transformation often acquire the capacity of proliferation and invasiveness and secrete more cell cycle associated proteins, such as proliferating cell nuclear antigen (PCNA) and Cyclin E (He et al., 2015). Traditionally, this process is triggered *via* the activation of the canonical transforming growth factor-β (TGF-β) pathway (Zhou Q. et al., 2016). However, peritoneal fibrosis has two cooperative parts, the fibrosis process itself and the inflammation (Zhou Q. et al., 2016; Balzer, 2020). The link between them is frequently bidirectional, with each one inducing the other (Balzer, 2020). Thus, the noncanonical inflammatory cytokines-elicited EMT also arouses the attention of researchers.

Particularly for IL-6, it is a multifunctional cytokine produced by a variety of cells such as lymphoid and non-lymphoid cells and by normal and transformed cells, including macrophages, mesothelial cells, and mesenchymal cells (Choy et al., 2020). The prospective clinic studies show that significant amounts of IL-6 in drained dialysate are in much higher concentrations than in serum under stable conditions (Lopes Barreto et al., 2011; Yang et al., 2014; Yang et al., 2018). The dialysate IL-6 level is increased shortly before the onset of and during the peritoneal fibrosis and several months after the clinically cured peritonitis, suggesting its local production and reflecting an intraperitoneal fibrosis and inflammatory state (Yang et al., 2014; Yang et al., 2018). However, the cellular mechanisms initiating an IL-6-related fibrosis response are still unclear. The current study aims to investigate the mechanism of IL-6-directed EMT, proliferation, and migration of MCs from an epigenetic point of view.

Epigenetics refers to heritable changes in gene expression which does not involve changes to the underlying DNA sequences (Guo et al., 2019). Acetylation is an important epigenetics modification in histone tail, which is regulated by histone acetyltransferases (HATs) and histone deacetylases (HDACs) (West and Johnstone, 2014). Histone deacetylase 6 (HDAC6) belongs to class IIb and primarily resides in the cytoplasm, while its deacetylase activity controls both cytoplasmic and nuclear functions (Pulya et al., 2021). The best characterized substrate for HDAC6 is α-tubulin (Hubbert et al., 2002). HDAC6 deacetylates α-tubulin *via* a process that requires its second HDAC domain and leads to an increase in the cell motility (Hubbert et al., 2002). Moreover, we and other research groups found that, unlike classes I, IIa, and III HDACs knockout mice, the homozygous HDAC6-deficient mice, which presented hyperacetylated tubulin in quite a lot tissues, were viable and fertile (Zhang et al., 2008; Chen et al., 2020). This indicated that HDAC6-specific inhibitors could be a safer and better tolerated medicine than pan-HDAC inhibitors. Recently, some of HDAC6 selective inhibitors, such as ACY-1215 (IC50 = 4.8 nM) and ACY-241 (IC50 = 2.6 nM), had been underwent the Phase I/II clinical trials in the field of tumors treatment (Porter et al., 2017; Pulya et al., 2021). Comparing these molecules, tubastatin A (TA) inhibited HDAC6 with an IC50 of 15 nM. Even so, TA was still a potential candidate because of its high selectivity. TA was found to have about 1093 fold selectivity towards HDAC6 over class Ⅰ HDACs, while ACY-1215 and ACY-241 had 12-fold and 18-fold selectivity, respectively (Porter et al., 2017; Pulya et al., 2021). Thus, TA was adopted and administrated at different concentrations in the current study.

Notably, previous studies reported that HDAC6 promoted kidney and lung fibrosis, mainly through its regulation and activation on growth factor receptors (Deribe et al., 2009; Saito et al., 2017; Chen et al., 2020). However, the role of HDAC6 in the inflammatory factor IL-6 signaling pathway remains unexplored. IL-6 was accounted for a significant concentration in the drained dialysate and thus adopted to stimulate human peritoneal mesothelial cells (HPMCs). Interestingly, we observed that HDAC6 was overexpressed after stimulation; meanwhile HPMCs tended to EMT, proliferation, and migration. What is the role of this IL-6-induced deacetylase for cell phenotype and mobility? Does it have a modification on the downstream signaling of IL-6? Could it be a linker between IL-6 and TGF-β signaling?

In this study, we first assessed the effect of TA on the IL-6-induced change of the phenotype and the mobility of HPMCs and further investigated the signaling regulatory mechanism, in order to provide evidence for future clinical trials in the field of peritoneal fibrosis.

## Materials and Methods

### Antibodies and Reagents

Tubastatin A and MG132 were purchased from Selleckchem (Houston, TX, United States). Antibodies to HDAC6 (#7612), Acetyl Histone H3 (Lys9) (#9649), Histone H3 (#9717), Acetyl α-Tubulin (Lys40) (#5335), α-Tubulin (#3873), ZO-1 (#13663), Smad3 (#9523), p-Smad3 (#9520), JAK2 (#3230), p-JAK2 (#3771), STAT3 (#9139), p-STAT3 (#9138), CTGF (#86641), E-cadherin (#14472), Cyclin E (#20808) and Snail (#3879) were purchased from Cell Signaling Technology (Danvers, MA, United States). Antibody to Fibronectin (ab2413) was purchased from Abcam (Cambridge, MA). Antibodies to GAPDH (sc-32233), Collagen I (A2) (sc-28654), TGFβRI (sc-399), Smad7 (sc-365846), and PCNA (sc-71858) were purchased from Santa Cruz Biotechnology (San Diego, CA, United States). IL-6 protein was purchased from R&D Systems (Minneapolis, MN, United States). HDAC6 siRNA was purchased from GenePharma (Shanghai, China). Lipofectamine 2000 was purchased from Invitrogen (Grand Island, NY, United States). The Cell Counting Kit-8 (CCK-8) proliferation assay kit was purchased from Beyotime Biotechnology (Haimen, China). Antibody to α-SMA (A2547) and all other chemicals were obtained from Sigma-Aldrich (St. Louis, MO, United States).

### Mesothelial Cell Culture

Human peritoneal mesothelial cells (HPMCs) (Jennio Biotechnology, Guangzhou, China) were cultured in MEM containing 10% fetal bovine serum (FBS) and 1% penicillin and streptomycin in an atmosphere of 5% CO_2_ and 95% air at 37°C. To determine the effect of HDAC6 inhibition on EMT induced by IL-6, the HPMCs were starved for 24 h with 0.5% FBS in MEM and then exposed to IL-6 (100 ng/ml) for 36 h in the presence or absence of different concentrations of TA (5, 10, and 20 µM) or MG132 (5 µM) before cell harvesting. All of the *in vitro* experiments were repeated for at least three times.

### siRNA Transfection

The small interfering (si) RNA oligonucleotides targeted specially for HDAC6 were used to downregulate the HDAC6 level in the cultured human peritoneal mesothelial cells. The HPMCs were seeded to 30–40% confluence in antibiotic-free medium and grown for 24 h and then were transfected with HDAC6 siRNA (50 nmol) with Lipofectamine 2000 according to the manufacturer’s instructions. In parallel, scrambled siRNA (50 nmol) was used as control for the off-target changes in the HPMCs. After transfection for 24 h, the cells were treated with IL-6 (100 ng/ml) for an additional 36 h before being harvested for further experiments.

### CCK-8 Proliferation Assay

The CCK-8 proliferation kit was used according to the manufacturer’s instructions. The HPMCs were starved for 24 h with MEM containing 0.5% FBS and then exposed to IL-6 (100 ng/ml) in the presence or absence of TA (5, 10, and 20 µM). After 36 h, the original culture medium was removed, and 100 µl new MEM medium containing 10 µl CCK-8 was added to each well in a 96-well plate for 37°C incubation for an additional 4 h. The final optical density values were read at 450 nm.

### Immunoprecipitations and Immunoblotting

Following an initial 24 h starvation, the HPMCs were exposed to IL-6 (100 ng/ml) for 36 h. The cell lysate was harvested by using ice-cold non-denaturing lysis buffer (Thermo Scientific, Rockford, IL). Co-immunoprecipitation (co-IP) was done using the Thermo Scientific Pierce co-IP kit (26149) following the manufacturer’s protocol. Briefly, the HDAC6 or IgG antibody was first immobilized for 2 h using AminoLink Plus coupling resin. The resin was then washed and incubated with the cell lysate overnight. After incubation, the resin was again washed and the protein was eluted using the elution buffer. The samples were analyzed by immunoblotting using the antibodies of Smad3, HDAC6, and GAPDH. Immunoblotting was performed as previously described (Zhou X. et al., 2016). The densitometry analysis of immunoblotting results was conducted by using ImageJ software.

### Wound Healing Assay

The HPMCs were seeded in a 6-well plate and allowed to reach 90% confluence. A scratch wound was created on the cell surface using a micropipette tip. Then, the cells were washed with PBS three times and incubated in serum-free MEM with IL-6 (100 ng/ml) in the presence or absence of TA (20 µM) and HDAC6 siRNA. The photomicrographs (× 40 objective magnification) of the migrating cells were taken at 0–36 h. The width of the wound was measured using ImageJ software (National Institutes of Health, Bethesda, MD, United States). The migratory rate was calculated as (A− B)/A × 100%, where A and B reflect the width of the wound at 0–36 h, respectively.

### Immunofluorescence Staining

The cells grown on chamber slides were fixed for 15 min with 4% paraformaldehyde. The samples were permeabilized with 0.1% Triton X-100 for 30 min. Then, the samples were blocked with goat serum for 15 min. The primary antibodies against HDAC6, Acetyl Histone H3, α-SMA, Fibronectin, E-cadherin, and ZO-1 diluted in PBS (1:100) were added to the samples, respectively, and incubated overnight at 4°C. After PBS washing, the cells were incubated with the Texas Red-labeled secondary antibody (1:200, Beyotime, China) diluted in PBS for 1 h at room temperature. The nuclei were stained with DAPI. After additional washing for 5 min three times, the samples were sealed by the antifade reagent and visualized with an Olympus fluorescence microscope (at ×200 magnification).

### Statistical Analysis

All the experiments were conducted at least three times. The data depicted in the graphs represent the means ± SEM for each group. The intergroup comparison was made using one-way analysis of variance. Multiple means were compared using Tukey’s test. The differences between the two groups were determined by Student’s *t*-test. The statistical significant difference between the mean values was marked in each graph. *p* < 0.05 was considered significant. The statistical analyses were conducted by using IBM SPSS Statistics 20.0 (Version X; IBM, Armonk, NY, United States).

## Results

### IL-6 Increases the Expression of HDAC6 in Cultured Peritoneal Mesothelial Cells

**FIGURE 1:**
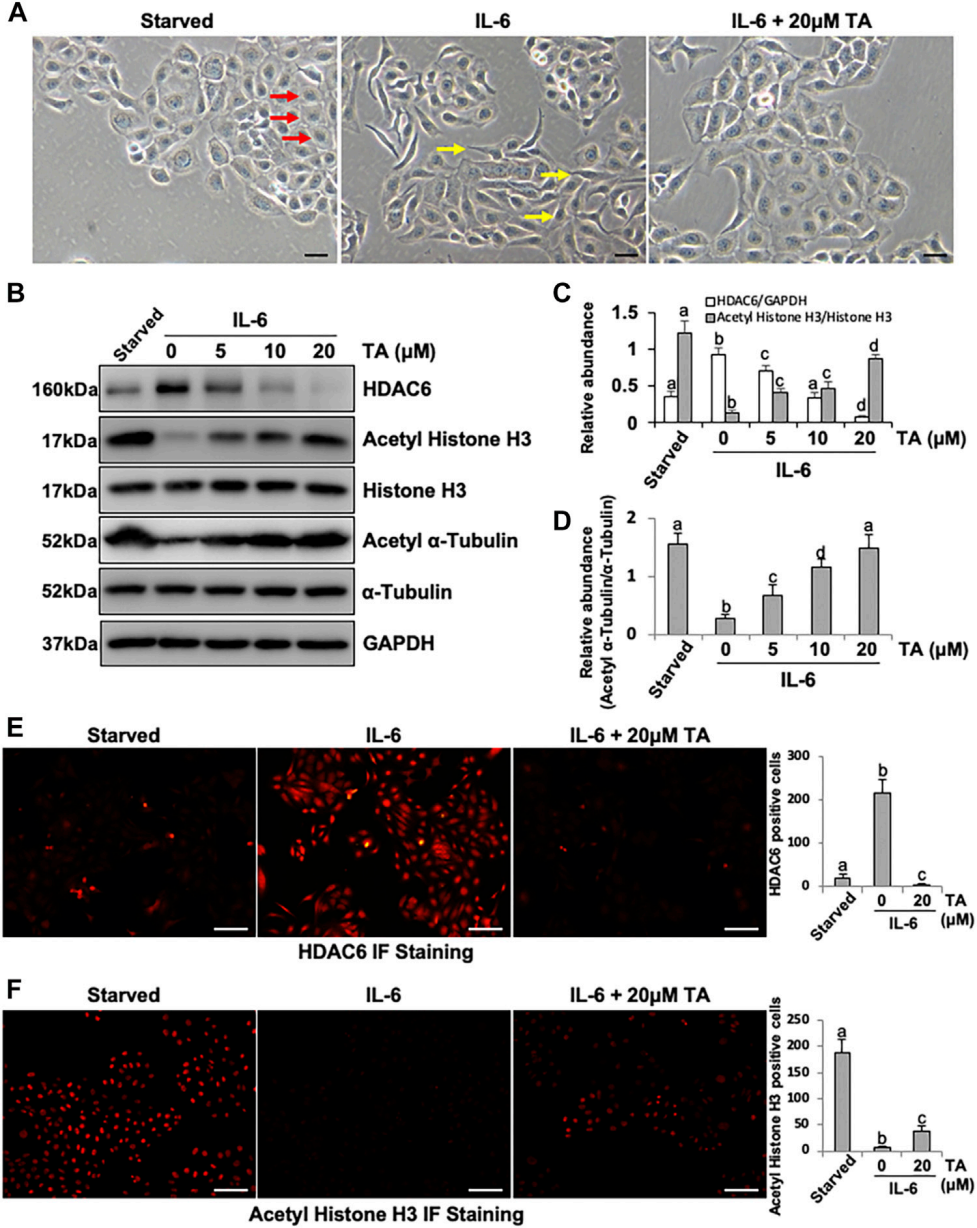
IL-6 increases the expression of HDAC6 in cultured human peritoneal mesothelial cells. **(A)** Cell morphology was observed using light microscopy. Red arrows indicate the cobblestone-shaped cells and yellow arrows show the spindle-shaped cells. **(B)** Cell lysates were subjected to immunoblot analysis with antibodies against HDAC6, Acetyl Histone H3, Histone H3, Acetyl α-Tubulin, α-Tubulin, and GAPDH. **(C)** Expression levels of HDAC6 and Acetyl Histone H3 were quantified by densitometry and normalized with GAPDH and total Histone H3, respectively. **(D)** Expression level of Acetyl α-Tubulin was quantified by densitometry and normalized with total α-Tubulin. Immunofluorescence photomicrographs (×200) illustrate staining of HDAC6 **(E)** and Acetyl Histone H3 **(F)**. In the HPMCs under different treatment, the count of HDAC6 or Acetyl Histone H3 positive cells was calculated from 10 random fields of each cell sample. Data are represented as the mean ± SEM (n = 3). Means with different superscript letters are significantly different from one another (*p* < 0.05). All scale bars = 100 μm.

IL-6 was a canonical inflammation factor, which was highly expressed in the fibrotic peritoneum and the dialysis effluent from long-term PD patients (Lopes Barreto et al., 2011; Yang et al., 2014; Yang et al., 2018). We aimed to investigate the mechanism by which IL-6 regulated the peritoneal fibrosis. Firstly, the human peritoneal mesothelial cells were stimulated by IL-6 *in vitro*. We found that the exposure of HPMCs to IL-6 at 100 ng/ml changed the cell morphology into fusiform or spindle shape (Figure 1A) and increased the expression level of HDAC6 and decreased the expression of Acetyl Histone H3 and Acetyl α-Tubulin in the cultured HPMCs (Figures 1B–D). The treatment of cells with TA, a highly selective inhibitor of HDAC6, at different concentrations (5, 10, and 20 μM) resulted in decreasing the expression of HDAC6 and increasing the expression of Acetyl Histone H3 (Figures 1B,C) and Acetyl α-Tubulin (Figures 1B,D) in a concentration-dependent manner, with a maximum effect at 20 μM. Neither IL-6 stimulation nor TA treatment had an impact on total expression of Histone H3 and α-Tubulin (Figure 1B). In addition, the immunofluorescent staining of HPMCs showed that HDAC6 was mainly expressed in both nucleus and the cytosol, while TA treatment decreased IL-6-induced HDAC6 and improved Acetyl Histone H3 (Figures 1E,F).

Notably, we found that TA not only inhibited the activity of HDAC6 but also reduced the expression of HDAC6. We suggested that TA induced the degradation of HDAC6 through the ubiquitin-proteasome pathway. It has been documented that MG132 is a specific inhibitor which can reduce the degradation of ubiquitin-conjugated proteins in mammalian cells (Longhitano et al., 2020). The HPMCs were exposed to IL-6 (100 ng/ml) and TA (20 μM) treatment in the presence or absence of MG132 (5 µM). Immunoblotting showed that TA significantly downregulated the expression of HDAC6, and further administration of MG132 resulted in upregulation of HDAC6 as indicated in Supplementary Figure S1. These data suggested that IL-6 increased the expression of HDAC6 in HPMCs, which was sensitive to TA and ubiquitination at least in part contributed to the downregulation of HDAC6.

### Pharmacological Blockade of HDAC6 Inhibits IL-6 Induced EMT of Cultured Human Peritoneal Mesothelial Cells

**FIGURE 2:**
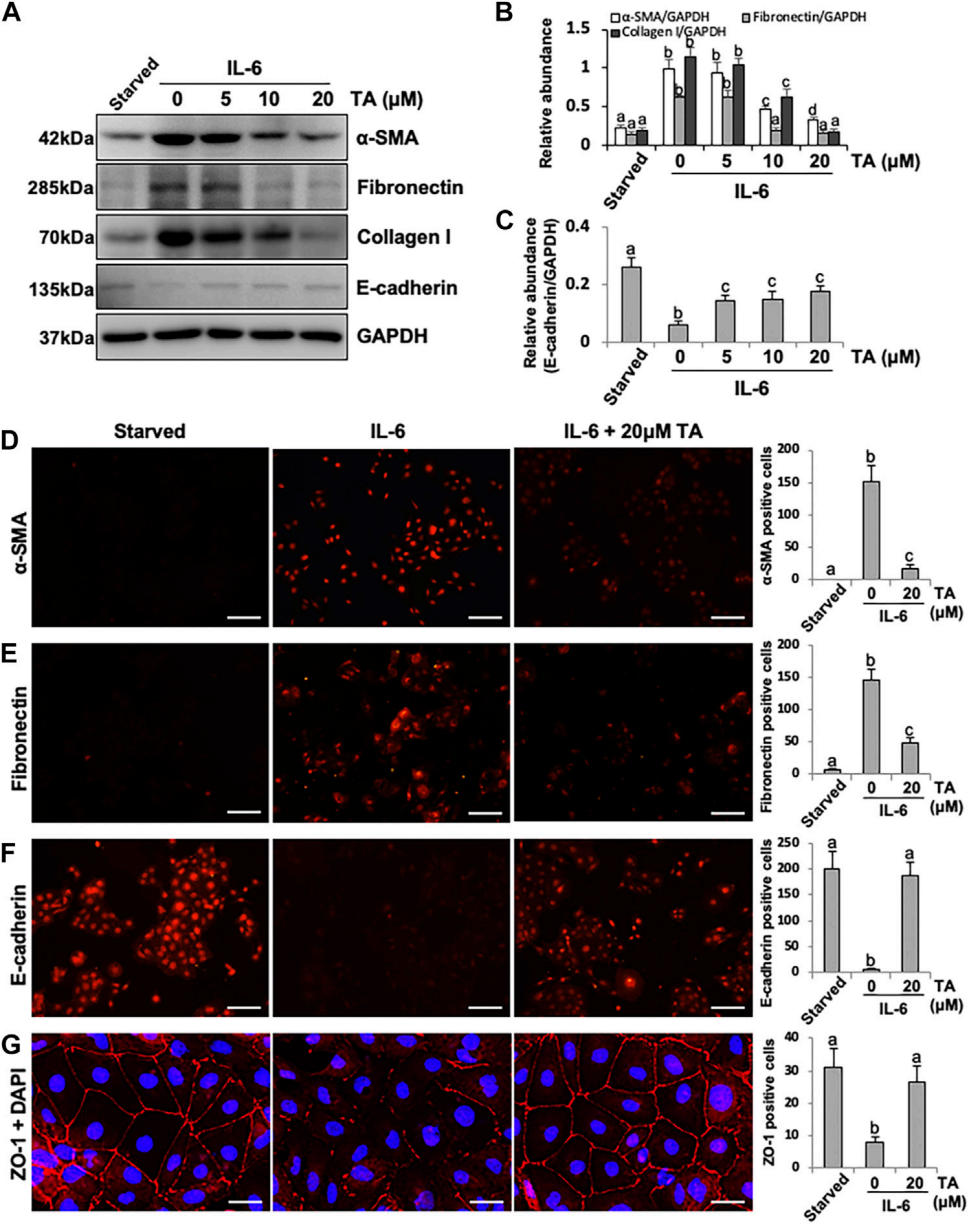
Pharmacological blockade of HDAC6 inhibits IL-6-induced EMT of cultured human peritoneal mesothelial cells. **(A)** Cell lysates were subjected to immunoblot analysis with specific antibodies against α-SMA, Fibronectin, Collagen I, E-cadherin, and GAPDH. **(B)** Expression levels of α-SMA, Fibronectin, and Collagen I were quantified by densitometry and normalized with GAPDH. **(C)** Expression level of E-cadherin was quantified by densitometry and normalized with GAPDH. Immunofluorescence photomicrographs (×200) illustrate staining of α-SMA **(D)**, Fibronectin **(E)**, E-cadherin **(F)**, and ZO-1 **(G)** in HPMCs under different treatment. The count of α-SMA, Fibronectin, E-cadherin, and ZO-1 positive cells was calculated from 10 random fields of each cell sample. Data are represented as the mean ± SEM (n = 3). Means with different superscript letters are significantly different from one another (*p* < 0.05). The scale bars in **D**, **E**, and **F** = 100 μm and the scale bar in **G** = 25 μm.

Beside the canonical TGF-β-directed EMT pathway, inflammatory cytokine IL-6 also facilitated the EMT of the peritoneal mesothelial cells (Xiao et al., 2017), while the specific mechanism was still obscure. Considering the high expression of HDAC6 under the IL-6 stimulation, we hypothesized that HDAC6 would play an essential role in IL-6-trigged EMT of HPMCs. To test this hypothesis, we examined the effect of HDAC6 inhibition on IL-6-induced EMT of peritoneal mesothelial cells. As shown in Figure 2, exposure to IL-6 promoted the EMT of HPMCs, showing the increased expression of mesenchymal cell markers (α-SMA, Fibronectin, and Collagen I) and the decreased expression of epithelial cell marker, E-cadherin (Strippoli et al., 2016). Inhibition of HDAC6 with TA markedly suppressed IL-6-induced expression of α-SMA, Fibronectin, and Collagen I and prevented E-cadherin loss (Figures 2A–C). In parallel, immunofluorescent staining had a similar phenomenon. TA treatment downregulated the expressions of α-SMA and Fibronectin and upregulated the expressions of E-cadherin and ZO-1 (Figures 2D–G). The results indicated that the inhibition of HDAC6 by TA could effectively alleviate IL-6-induced EMT of HPMCs.

### siRNA-Mediated Silencing of HDAC6 Inhibits EMT of Peritoneal Mesothelial Cells

**FIGURE 3:**
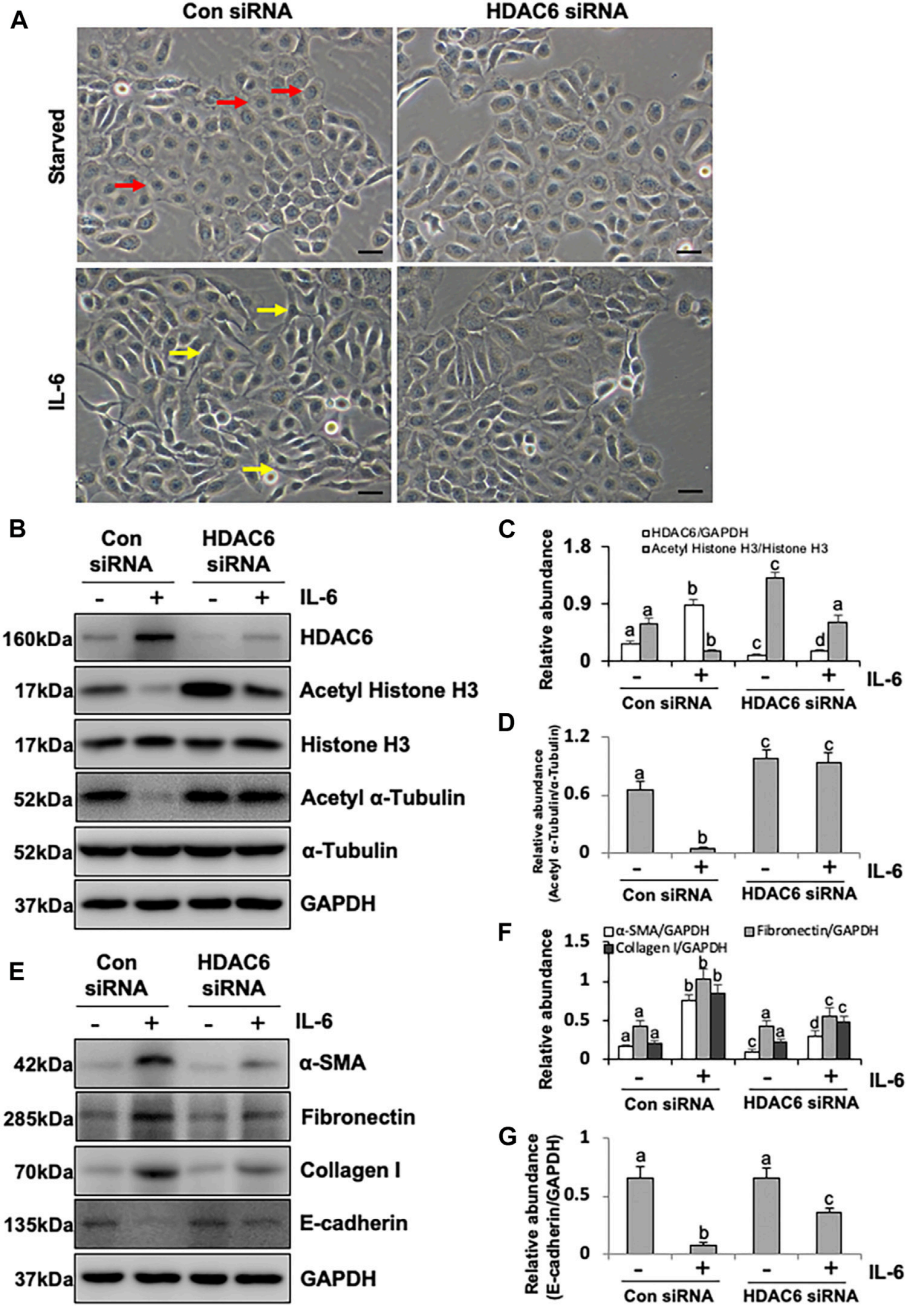
siRNA-medicated silencing of HDAC6 inhibits EMT of peritoneal mesothelial cells. **(A)** Cell morphology was observed using light microscopy. The red arrows indicated the cobblestone-shaped cells and the yellow arrows showed the spindle-shaped cells. **(B)** Cell lysates were subjected to immunoblot analysis with antibodies against HDAC6, Acetyl Histone H3, Histone H3, Acetyl α-Tubulin, α-Tubulin, and GAPDH. **(C)** Expression levels of HDAC6 and Acetyl Histone H3 were quantified by densitometry and normalized with GAPDH and total histone H3, respectively. **(D)** Expression level of Acetyl α-Tubulin was quantified by densitometry and normalized with total α-Tubulin. **(E)** Cell lysates were subjected to immunoblot analysis with specific antibodies against α-SMA, Fibronectin, Collagen I, E-cadherin, and GAPDH. **(F)** Expression levels of α-SMA, Fibronectin, and Collagen I were quantified by densitometry and normalized with GAPDH. **(G)** Expression level of E-cadherin was quantified by densitometry and normalized with GAPDH. Data are represented as the mean ± SEM (n = 3). Means with different superscript letters are significantly different from one another (*p* < 0.05). All scale bars = 100 μm.

To further verify the role of HDAC6 in EMT, we tested the effect of HDAC6 knockdown on the EMT of HPMCs using specific siRNA. As shown in Figure 3A, the cell morphology was slightly turned into oval or cobblestone shape after HDAC6 inhibition. siRNA-mediated silencing of HDAC6 recovered the abnormally low expression of Acetyl Histone H3 and Acetyl α-Tubulin induced by IL-6 but did not alter the level of total Histone H3 and α-Tubulin (Figures 3B–D). As expected, HDAC6 siRNA also downregulated α-SMA, Fibronectin, and Collagen I and upregulated the expression of E-cadherin (Figures 3E–G). These data further confirmed the importance of HDAC6 in mediating IL-6 induced EMT of peritoneal mesothelial cells.

### HDAC6 Is Required for the Activation of the TGF-β Signaling Pathway in Peritoneal Mesothelial Cells

**FIGURE 4:**
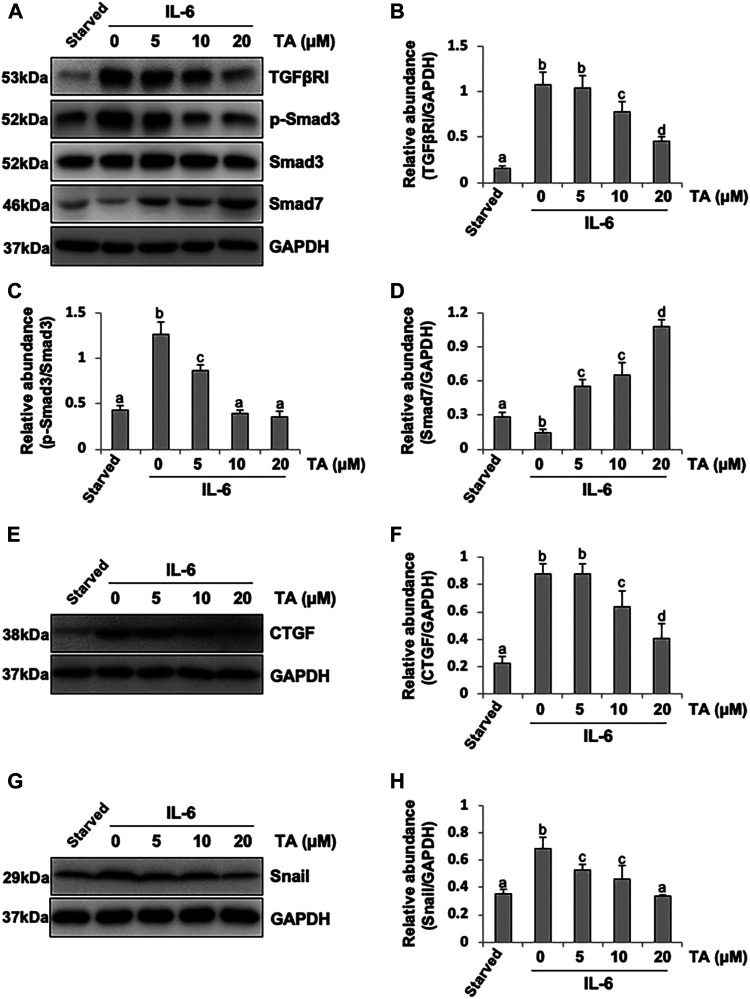
Pharmacological blockade of HDAC6 inhibits the activation of the TGF-β signaling pathway and decreases the expression of CTGF and Snail in peritoneal mesothelial cells. **(A)** Cell lysates were subjected to immunoblot analysis with specific antibodies against TGFβRI, p-Smad3, Smad3, Smad7, and GAPDH. **(B)** Expression level of TGFβRI was quantified by densitometry and normalized with GAPDH. **(C)** Expression level of p-Smad3 was quantified by densitometry and normalized with Smad3. **(D)** Expression level of Smad7 was quantified by densitometry and normalized with GAPDH. **(E)** Cell lysates were subjected to immunoblot analysis with specific antibodies against CTGF and GAPDH. **(F)** Expression level of CTGF was quantified by densitometry and normalized with GAPDH. **(G)** Cell lysates were subjected to immunoblot analysis with specific antibodies against Snail and GAPDH. **(H)** Expression level of Snail was quantified by densitometry and normalized with GAPDH. Data are represented as the mean ± SEM (n = 3). Means with different superscript letters are significantly different from one another (*p* < 0.05).

**FIGURE 5:**
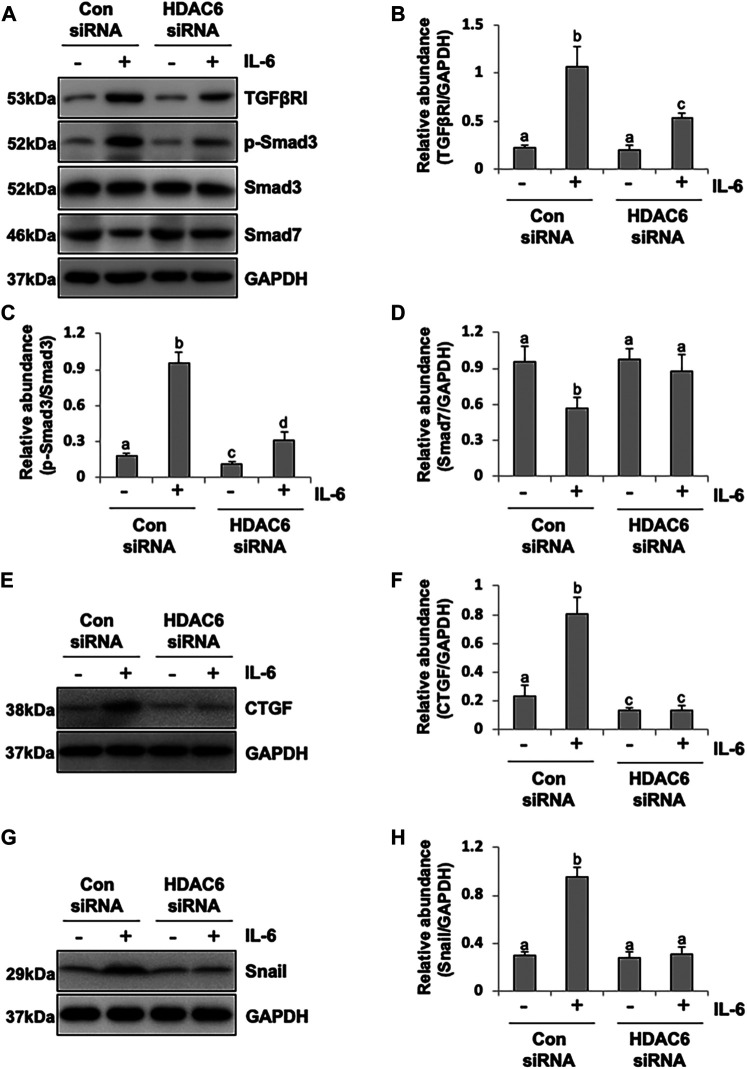
siRNA-medicated silencing of HDAC6 inhibits the activation of the TGF-β signaling pathway and decreases the expression of CTGF and Snail in peritoneal mesothelial cells. **(A)** Cell lysates were subjected to immunoblot analysis with specific antibodies against TGFβRI, p-Smad3, Smad3, Smad7, and GAPDH. **(B)** Expression level of TGFβRI was quantified by densitometry and normalized with GAPDH. **(C)** Expression level of p-Smad3 was quantified by densitometry and normalized with Smad3. **(D)** Expression level of Smad7 was quantified by densitometry and normalized with GAPDH. **(E)** Cell lysates were subjected to immunoblot analysis with specific antibodies against CTGF and GAPDH. **(F)** Expression level of CTGF was quantified by densitometry and normalized with GAPDH. **(G)** Cell lysates were subjected to immunoblot analysis with specific antibodies against Snail and GAPDH. **(H)** Expression level of Snail was quantified by densitometry and normalized with GAPDH. Data are represented as the mean ± SEM (n = 3). Means with different superscript letters are significantly different from one another (*p* < 0.05).

It was reported that IL-6 could trigger the activation of the TGF-β signaling pathway in the fibrosis process (Luckett-Chastain et al., 2017); however, the cross-talk mechanism was obscure. We speculated that IL-6-elicited HDAC6 was required for the activation of the TGF-β signaling pathway, leading to an increased secretion of growth factors, such as CTGF, and the increased expression of transcription factors, such as Snail. To test this hypothesis, we examined the expression of TGF-β type receptor I (TGFβRI) on the TA-inhibited peritoneal mesothelial cells. As shown in Figures 4A,B, the base level of TGFβRI was negligible in the starved cell. However, its expression level was substantially elevated after IL-6 stimulation, which was further inhibited by TA treatment in a dose-dependent manner. TA also suppressed the phosphorylation of Smad3, while it restored the expression of Smad7, which could block the activation of Smad2/3 by competitively binding with TGFβRI (Murayama et al., 2020). There was no impact on total Smad3 (Figures 4A–D). Moreover, the immunoblot results presented that TA decreased the secretion of the growth factor CTGF in the IL-6 stimulated HPMCs (Figures 4E,F); and the nuclear transcription factor Snail, which was responsible for transcription inhibition of E-cadherin (Zhang et al., 2018; Wang et al., 2020), also witnessed a decrease after TA treatment (Figures 4G,H). Similarly, HDAC6 siRNA inhibited accumulation of TGFβRI, phosphorylation of Smad3, and loss of Smad7 (Figures 5A–D). It also had an inhibition effect on the expressions of CTGF and Snail (Figures 5E–H). These results suggested that HDAC6 was required for the activation of the TGF-β/Smad3 signaling pathway, resulting in the secretion of CTGF and upregulation of Snail (Figure 8).

Additionally, we conducted the immunoprecipitation experiment to find the direct substrate of HDAC6 (Supplementary Figure S2). The starved HPMCs were exposed to IL-6 (100 ng/ml) for 36 h and then harvested and prepared for immunoprecipitation and immunoblot. The result showed an interaction between HDAC6 and Smad3 in HPMCs treated with IL-6. Therefore, we speculated that HDAC6 directly interacted with Smad3 in the cytoplasm, and deacetylated Smad3 tended to nuclear localization and phosphorylation.

### Inhibition of HDAC6 with TA or siRNA Alleviates Proliferation and Migration Through the JAK2/STAT3 Signaling Pathway in HPMCs.

**FIGURE 6:**
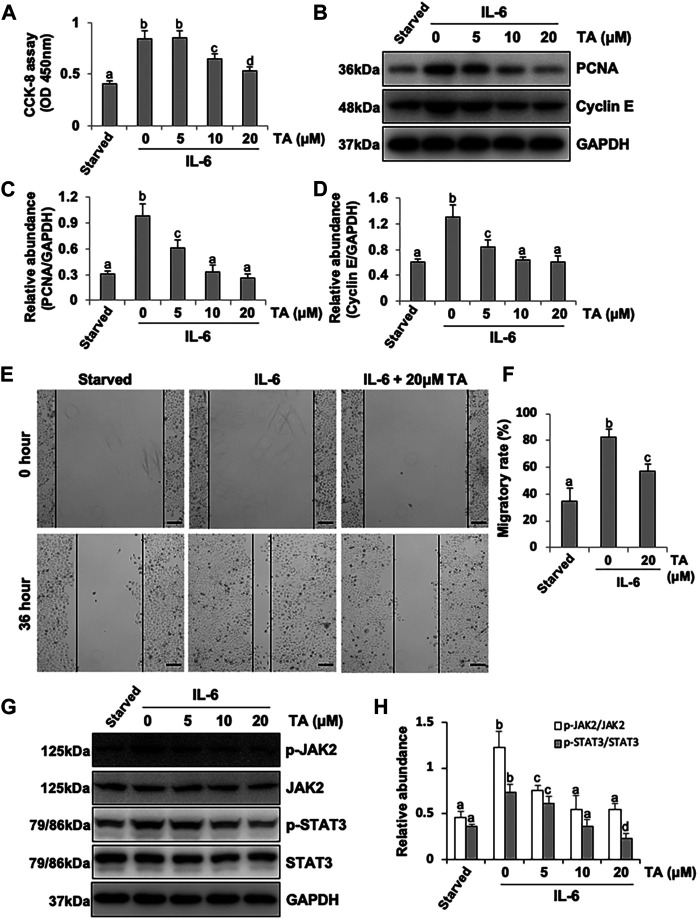
Pharmacological blockade of HDAC6 alleviates proliferation and migration through the JAK2/STAT3 signaling pathway in human peritoneal mesothelial cells. **(A)** Cell proliferation was assessed by the CCK-8 assay. **(B)** Cell lysates were subjected to the immunoblot analysis with specific antibodies against PCNA, Cyclin E, and GAPDH. **(C)** Expression level of PCNA was quantified by densitometry and normalized with GAPDH. **(D)** Expression level of Cyclin E was quantified by densitometry and normalized with GAPDH. **(E)** Wound-healing assay of HPMCs treated with IL-6 (100 ng/ml) in the presence or absence of TA (20 μM). Photomicrographs of migrating cells were taken at 0 and 36 h. **(F)** The width of the wound was measured, and the migratory rate was calculated. **(G)** Cell lysates were subjected to the immunoblot analysis with specific antibodies against p-JAK2, JAK2, p-STAT3, STAT3, and GAPDH. **(H)** Expression levels of p-JAK2 and p-STAT3 were quantified by densitometry and normalized with JAK2 and STAT3, respectively. Data are represented as the mean ± SEM (n = 3). Means with different superscript letters are significantly different from one another (*p* < 0.05). All scale bars = 500 μm.

**FIGURE 7:**
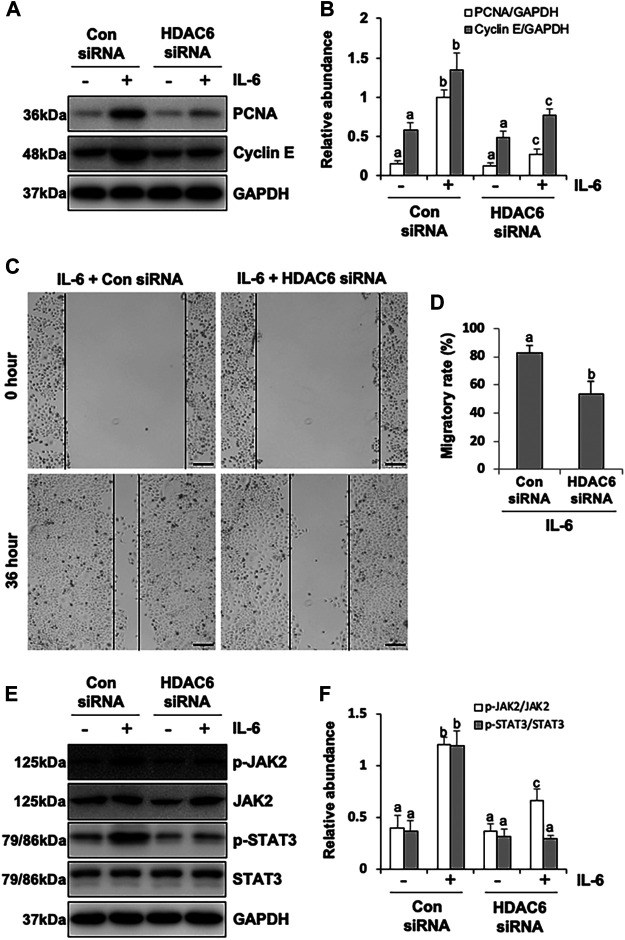
siRNA-medicated silencing of HDAC6 inhibits proliferation and migration through the JAK2/STAT3 signaling pathway in human peritoneal mesothelial cells. **(A)** Cell lysates were subjected to the immunoblot analysis with specific antibodies against PCNA, Cyclin E, and GAPDH. **(B)** Expression levels of PCNA and Cyclin E were quantified by densitometry and normalized with GAPDH. **(C)** The wound-healing assay of the HPMCs treated with IL-6 (100 ng/ml) in the presence of HDAC6 siRNA or scrambled siRNA. Photomicrographs of migrating cells were taken at 0 and 36 h. **(D)** The width of the wound was measured, and the migratory rate was calculated. **(E)** Cell lysates were subjected to the immunoblot analysis with specific antibodies against p-JAK2, JAK2, p-STAT3, STAT3, and GAPDH. **(F)** Expression levels of p-JAK2 and p-STAT3 were quantified by densitometry and normalized with JAK2 and STAT3, respectively. Data are represented as the mean ± SEM (n = 3). Means with different superscript letters are significantly different from one another (*p* < 0.05). All scale bars = 500 μm.

**FIGURE 8:**
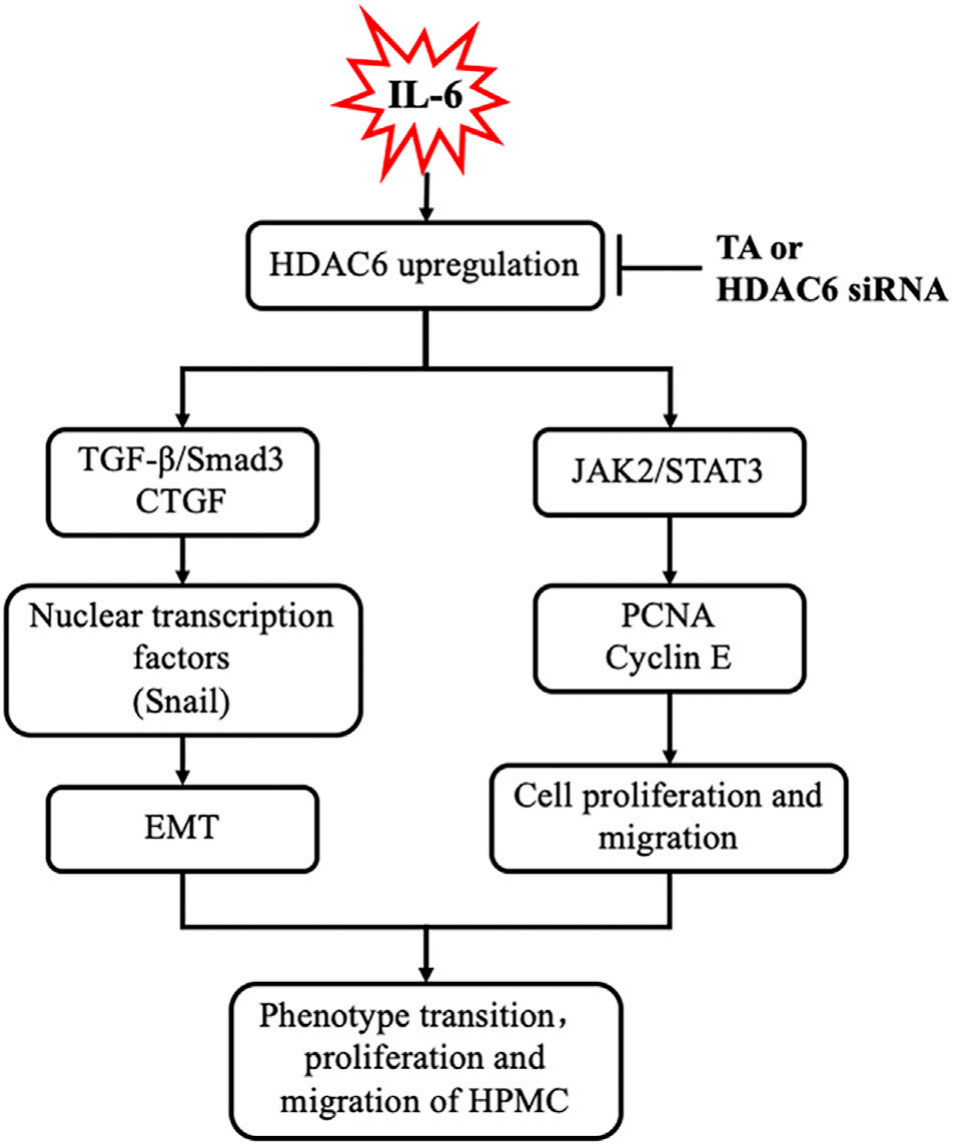
Requirement of HDAC6 for IL-6 induced EMT, proliferation, and migration of peritoneal mesothelial cells resulted in an aggravation of peritoneal fibrosis. Exposure of IL-6 to the peritoneal mesothelial cells upregulates HDAC6, which leads to activation of TGF-β/Smad3 and JAK2/STAT3 signaling, subsequently inducing EMT (i.e., expression of CTGF and Snail), proliferation, and migration (i.e., production of PCNA and Cyclin E) response. All these responses are inhibited by TA or HDAC6 siRNA.

It was reported that HPMCs underwent EMT acquired the ability of cell cycle progression, cell proliferation, and cell mobility (He et al., 2015). Thus, we assessed the role of HDAC6 in regulating the proliferation of HPMCs by the CCK-8 assay. The results showed that IL-6 at 100 ng/ml substantially stimulated the cell proliferation of HPMCs, compared with the simple starved group, while the TA treatment kept down the proliferation level, especially at the concentration of 20 μM (Figure 6A). The immunoblot analysis further confirmed the ability of TA in inhibiting cell proliferation, as indicated by decreased expression levels of PCNA and Cyclin E, two proliferating hallmarks (Figures 6B–D). In addition, the cell migration test showed that TA suppressed the migratory rate of HPMCs (Figures 6E,F). To further reveal the mechanism in TA-downregulated proliferation and migration, we tested the expressions of JAK2 and STAT3 before and after the TA treatment in IL-6-stimulated HPMCs. The immunoblot analysis showed that TA suppressed the phosphorylation of JAK2 and STAT3 in a concentration-dependent manner, while the total protein was not impacted (Figures 6G,H). Additionally, we found that siRNA-mediated silencing of HDAC6 achieved similar results (Figure 7). Taken together, these data illustrated that HDAC6 promoted the proliferation and migration of HPMCs through regulating the JAK2/STAT3 signaling (Figure 8).

## Discussion

The mesothelial cells which transferred to the mesenchymal phenotype acquired the increased capacity of proliferation, migration, and extracellular matrix (ECM) secretion, forming a fibrosis scar (Zhou Q. et al., 2016). Beside the canonical TGF-β, the inflammation factor IL-6 was also an important fibrosis inducer, which had a significant amount in the drained dialysate from the PD patients (Lopes Barreto et al., 2011; Yang et al., 2014; Yang et al., 2018). However, the specific mechanism in the IL-6-induced cell phenotype change was still obscure. The current study firstly unravels a pivotal role of IL-6-elicited HDAC6 in signaling regulation and in EMT, proliferation, and migration of HPMCs.

It had been well established that TGF-β1 triggers EMT. Indeed, IL-6 also had this capacity, which was an indirect process through coactivating the TGF-β signaling pathway (Luckett-Chastain et al., 2017). Although the IL-6/TGF-β cooperation in the realm of ECM deposition had been documented (Luckett-Chastain et al., 2017; Epstein Shochet et al., 2020), their modulatory link was obscure. Our current study found that IL-6 stimulation induced the overexpression of HDAC6 in HPMCs. HDAC6 might be a vital linker between two signalings. The best characterized substrate for HDAC6 was α-Tubulin, an important component of the cytoskeleton, which had been reported to closely contact with clathrin, a receptor endocytosis-related protein (Rappoport et al., 2003; Montagnac et al., 2013). Evidence showed that clathrin moved along the microtubule cytoskeleton parallel to the cell surface (Rappoport et al., 2003; Montagnac et al., 2013). Acetylation of α-Tubulin tended to form a polymerization of microtubule and resulted in modest reduced endocytosis, while deacetylated α-Tubulin facilitated the insertion of the complexes in the clathrin-coated pits (Thomas et al., 2016; Melgari et al., 2020). Given that the TGF-β receptors were constitutively internalized *via* clathrin-dependent or lipid raft-dependent endocytic pathways (Kardassis et al., 2009), we suggested that IL-6 might modulate the activity of TGFβRⅠ through upregulating deacetylase HDAC6, deacetylating α-Tubulin, and subsequent promoting clathrin internalization. This was evidenced by our present data demonstrating that HDAC6 inhibition increased the acetylation of α-Tubulin, decreased the expression of TGFβRⅠ, and the activation of the TGF-β network in IL-6 treated HPMCs. But how does IL-6 increase the expression and activity of HDAC6? It might also work through an epigenetic mechanism. The detailed mechanism waited further investigation.

Our study also found that HDAC6 affected the activity of Smad3 in IL-6-treated HPMCs. The activation of Smad3 was not just the downstream effector of the TGF receptor. The emerging evidence implicated that the microtubule structure regulated the Smads activity (Dong et al., 2000; Zhu et al., 2004). Binding of Smads to microtubules kept Smads in their inactive stage, and TGF-β1 could trigger the release of Smads from microtubules and the subsequent phosphorylation of Smads (Dong et al., 2000; Zhu et al., 2004). Thus, one possible mechanism is that HDAC6 modulated the Smad3 activity through deacetylating α-Tubulin and promoting the release of Smad3. Another mechanism of Smad3 activation might be a direct acetylation modification by HDAC6 itself. A recent study reported the requirement of HDAC6 for the epidermal growth factor- (EGF-) triggered nuclear localization of β-catenin (Li et al., 2008). The deacetylation of lysine 49 in β-catenin was suggested to be essential for nuclear localization of β-catenin. It was conceivable that HDAC6 regulated nuclear localization of Smad3 *via* deacetylating Smad3 or Smad3-interacting proteins. As expected, the immunoprecipitation experiment confirmed a direct interaction between HDAC6 and Smad3 in HPMCs treated with IL-6. Subsequently, intranuclear Smad3 recruited and activated the transcription factor Snail and promoted the expression of the ECM protein and the secretion of CTGF. Our results showed that HDAC6 was required for the phosphorylation of Smad3, while it did not affect the total expression of Smad3. Interestingly, Smad7, a Smad3 blocker in the TGF-β network, encountered a significant increase following the HDAC6 inhibition. Collectively, we suggested that HDAC6 was required for IL-6-elicited TGF-β signaling transduction through upregulation of TGFβRⅠ, activation of Smad3, and downregulation of Smad7. Nevertheless, the mechanisms underlying HDAC6-mediated Smad signaling need further investigation.

Another important transcription factor in IL-6 signaling was STAT3, which was responsible for cell proliferation and invasion (Cheng et al., 2020). We speculated that activation of JAK2/STAT3 might relate to deacetylation of α-Tubulin. Like many other transcription factors, STAT3 possessed a coiled-coil domain, which was found in a variety of tubulin-binding proteins (Zhang et al., 2000; Tala et al., 2014). In leukemia cells, STAT3 was mainly concentrated in the centrosome region where microtubules were more stable and hyperacetylated (Yan et al., 2015). It was possible that deacetylation of α-Tubulin promoted the activation and nuclear translocation of STAT3, which might generate a positive feedback and affect phosphorylation of JAK2, contributing to an amplifying IL-6/JAK2/STAT3 signaling. Thus, the cell cycle associated proteins (i.e., PCNA and Cyclin E) increased and resulted in cell proliferation. Moreover, HDAC6 also promoted the cell migration. It was documented that the interaction between deacetylated α-Tubulin and STAT3 changed the stability of microtubule and activated the small GTPase Rac-1 or Rho to control actin-dependent membrane ruffling and cell motility (Hubbert et al., 2002). Consistently, HDAC6 inhibition reduced the STAT3 response and blocked the feedback for JAK2, slowing down the proliferation and migration of HPMCs.

In conclusion, the current study firstly found that IL-6-induced deacetylase HDAC6 not only regulated IL-6 itself downstream the JAK2/STAT3 signaling but also co-activated the TGF-β/Smad3 signaling, leading to the change of the phenotype and mobility of cells. This finding partially interpreted the mechanism of the development of peritoneal fibrosis under the inflammation microenvironment. HDAC6 could be a potential therapeutic target for the prevention and treatment of peritoneal fibrosis.

## Data Availability

The original contributions presented in the study are included in the article/Supplementary Material; further inquiries can be directed to the corresponding author.

## Supplementary Material

Supplementary Figure S1 TA induces the degradation of HDAC6 through ubiquitin-proteasome pathway. **(A)** Cell lysates are subjected to immunoblot analysis with specific antibodies against HDAC6 and GAPDH. **(B)** Expression level of HDAC6 is quantified by densitometry and normalized with GAPDH. Data are represented as the mean ± S.E.M (*n* = 3). Means with different superscript letters are significantly different from one another (*p* < 0.05).

Supplementary Figure S2 Smad3 is identified as a direct substrate of HDAC6. **(A)** Following an initial 24-h starvation, HPMCs are exposed to IL-6 (100 ng/ml) for 36 h. Total cellular proteins are harvested and immunoprecipitations are performed using HDAC6 or IgG antibody. And then immunoprecipitated proteins are immunoblotted with specific antibodies against Smad3, HDAC6 and GAPDH. An interaction between HDAC6 and Smad3 is found in HPMCs treated with IL-6.

## Abbreviations

HPMCs, human peritoneal mesothelial cells; HDAC6, histone deacetylase 6; IL-6, interleukin-6; EMT, epithelial-mesenchymal transition; TA, tubastatin A; α-SMA, α-smooth muscle actin; TGFβ, transforming growth factor β, TGFβRI; TGF-β receptor I; CTGF, connective tissue growth factor; PD, peritoneal dialysis; PF, peritoneal fibrosis.

## References

[B1] Balzer M. S. (2020). Molecular Pathways in Peritoneal Fibrosis. Cell Signal. 75, 109778. 10.1016/j.cellsig.2020.109778 32926960

[B2] Chen X. Yu C. Hou X. Li J. Li T. Qiu A. (2020). Histone Deacetylase 6 Inhibition Mitigates Renal Fibrosis by Suppressing TGF-β and EGFR Signaling Pathways in Obstructive Nephropathy. Am. J. Physiology-Renal Physiol. 319 (6), F1003–f1014. 10.1152/ajprenal.00261.2020 PMC7792693 33103445

[B3] Cheng M. Liu P. Xu L. X. (2020). Iron Promotes Breast Cancer Cell Migration via IL-6/JAK2/STAT3 Signaling Pathways in a Paracrine or Autocrine IL-6-rich Inflammatory Environment. J. Inorg. Biochem. 210, 111159. 10.1016/j.jinorgbio.2020.111159 32652260

[B4] Choy E. H. De Benedetti F. Takeuchi T. Hashizume M. John M. R. Kishimoto T. (2020). Translating IL-6 Biology into Effective Treatments. Nat. Rev. Rheumatol. 16 (6), 335–345. 10.1038/s41584-020-0419-z 32327746 PMC7178926

[B5] Deribe Y. L. Wild P. Chandrashaker A. Curak J. Schmidt M. H. H. Kalaidzidis Y. (2009). Regulation of Epidermal Growth Factor Receptor Trafficking by Lysine Deacetylase HDAC6. Sci. Signal. 2 (102), ra84. 10.1126/scisignal.2000576 20029029

[B6] Dong C. Li Z. Alvarez R. Jr. Feng X.-H. Goldschmidt-Clermont P. J. (2000). Microtubule Binding to Smads May Regulate TGFβ Activity. Mol. Cel. 5 (1), 27–34. 10.1016/s1097-2765(00)80400-1 10678166

[B7] Epstein Shochet G. Brook E. Bardenstein-Wald B. Shitrit D. (2020). TGF-β Pathway Activation by Idiopathic Pulmonary Fibrosis (IPF) Fibroblast Derived Soluble Factors Is Mediated by IL-6 Trans-signaling. Respir. Res. 21 (1), 56. 10.1186/s12931-020-1319-0 32070329 PMC7029598

[B8] Guo C. Dong G. Liang X. Dong Z. (2019). Epigenetic Regulation in AKI and Kidney Repair: Mechanisms and Therapeutic Implications. Nat. Rev. Nephrol. 15 (4), 220–239. 10.1038/s41581-018-0103-6 30651611 PMC7866490

[B9] He L. Che M. Hu J. Li S. Jia Z. Lou W. (2015). Twist Contributes to Proliferation and Epithelial-To-Mesenchymal Transition-Induced Fibrosis by Regulating YB-1 in Human Peritoneal Mesothelial Cells. Am. J. Pathol. 185 (8), 2181–2193. 10.1016/j.ajpath.2015.04.008 26055210

[B10] Hubbert C. Guardiola A. Shao R. Kawaguchi Y. Ito A. Nixon A. (2002). HDAC6 Is a Microtubule-Associated Deacetylase. Nature 417 (6887), 455–458. 10.1038/417455a 12024216

[B11] Kardassis D. Murphy C. Fotsis T. Moustakas A. Stournaras C. (2009). Control of Transforming Growth Factor β Signal Transduction by Small GTPases. Febs j 276 (11), 2947–2965. 10.1111/j.1742-4658.2009.07031.x 19490100

[B12] Lamouille S. Xu J. Derynck R. (2014). Molecular Mechanisms of Epithelial-Mesenchymal Transition. Nat. Rev. Mol. Cel Biol. 15 (3), 178–196. 10.1038/nrm3758 PMC4240281 24556840

[B13] Li P. K.-T. Chow K. M. Van De Luijtgaarden M. W. M. Johnson D. W. Jager K. J. Mehrotra R. (2017). Changes in the Worldwide Epidemiology of Peritoneal Dialysis. Nat. Rev. Nephrol. 13 (2), 90–103. 10.1038/nrneph.2016.181 28029154

[B14] Li Y. Zhang X. Polakiewicz R. D. Yao T.-P. Comb M. J. (2008). HDAC6 Is Required for Epidermal Growth Factor-Induced β-Catenin Nuclear Localization. J. Biol. Chem. 283 (19), 12686–12690. 10.1074/jbc.C700185200 18356165 PMC3762558

[B15] Longhitano L. Tibullo D. Giallongo C. Lazzarino G. Tartaglia N. Galimberti S. (2020). Proteasome Inhibitors as a Possible Therapy for SARS-CoV-2. Ijms 21 (10), 3622. 10.3390/ijms21103622 PMC7279248 32443911

[B16] Lopes Barreto D. Coester A. M. Noordzij M. Smit W. Struijk D. G. Rogers S. (2011). Variability of Effluent Cancer Antigen 125 and Interleukin-6 Determination in Peritoneal Dialysis Patients. Nephrol. Dial. Transplant. 26 (11), 3739–3744. 10.1093/ndt/gfr170 21498425

[B17] Luckett-Chastain L. R. Cottrell M. L. Kawar B. M. Ihnat M. A. Gallucci R. M. (2017). Interleukin (IL)-6 Modulates Transforming Growth Factor-β Receptor I and II (TGF-Βri and II) Function in Epidermal Keratinocytes. Exp. Dermatol. 26 (8), 697–704. 10.1111/exd.13260 27892604 PMC5446936

[B18] Melgari D. Barbier C. Dilanian G. Rücker-Martin C. Doisne N. Coulombe A. (2020). Microtubule Polymerization State and Clathrin-dependent Internalization Regulate Dynamics of Cardiac Potassium Channel. J. Mol. Cell Cardiol. 144, 127–139. 10.1016/j.yjmcc.2020.05.004 32445844

[B19] Montagnac G. Meas-Yedid V. Irondelle M. Castro-Castro A. Franco M. Shida T. (2013). αTAT1 Catalyses Microtubule Acetylation at Clathrin-Coated Pits. Nature 502 (7472), 567–570. 10.1038/nature12571 24097348 PMC3970258

[B20] Murayama K. Kato-Murayama M. Itoh Y. Miyazono K. Miyazawa K. Shirouzu M. (2020). Structural Basis for Inhibitory Effects of Smad7 on TGF-β Family Signaling. J. Struct. Biol. 212 (3), 107661. 10.1016/j.jsb.2020.107661 33166654

[B21] Porter N. J. Mahendran A. Breslow R. Christianson D. W. (2017). Unusual Zinc-Binding Mode of HDAC6-Selective Hydroxamate Inhibitors. Proc. Natl. Acad. Sci. USA. 114 (51), 13459–13464. 10.1073/pnas.1718823114 29203661 PMC5754815

[B22] Pulya S. Amin S. A. Adhikari N. Biswas S. Jha T. Ghosh B. (2021). HDAC6 as Privileged Target in Drug Discovery: A Perspective. Pharmacol. Res. 163, 105274. 10.1016/j.phrs.2020.105274 33171304

[B23] Rappoport J. Z. Taha B. W. Simon S. M. (2003). Movement of Plasma-Membrane-Associated Clathrin Spots along the Microtubule Cytoskeleton. Traffic 4 (7), 460–467. 10.1034/j.1600-0854.2003.00100.x 12795691

[B24] Saito S. Zhuang Y. Shan B. Danchuk S. Luo F. Korfei M. (2017). Tubastatin Ameliorates Pulmonary Fibrosis by Targeting the TGFβ-Pi3k-Akt Pathway. PLoS One 12 (10), e0186615. 10.1371/journal.pone.0186615 29045477 PMC5646855

[B25] Strippoli R. Moreno-Vicente R. Battistelli C. Cicchini C. Noce V. Amicone L. (2016). Molecular Mechanisms Underlying Peritoneal EMT and Fibrosis. Stem Cell Int. 2016, 1–11. 10.1155/2016/3543678 PMC4752998 26941801

[B26] Tala Sun X. Chen J. Zhang L. Liu N. Zhou J. (2014). Microtubule Stabilization by Mdp3 Is Partially Attributed to its Modulation of HDAC6 in Addition to its Association with Tubulin and Microtubules. PLoS One 9 (3), e90932. 10.1371/journal.pone.0090932 24614595 PMC3948737

[B27] Thomas G. W. Rael L. T. Hausburg M. Frederick E. D. Brody E. Bar-Or D. (2016). The Low Molecular Weight Fraction of Commercial Human Serum Albumin Induces Acetylation of α-tubulin and Reduces Transcytosis in Retinal Endothelial Cells. Biochem. Biophysical Res. Commun. 478 (4), 1780–1785. 10.1016/j.bbrc.2016.09.026 27613088

[B28] Wang H. Li Q.-F. Chow H. Choi S. Leung Y.-C. (2020). Arginine Deprivation Inhibits Pancreatic Cancer Cell Migration, Invasion and EMT via the Down Regulation of Snail, Slug, Twist, and MMP1/9. J. Physiol. Biochem. 76 (1), 73–83. 10.1007/s13105-019-00716-1 31823303

[B29] West A. C. Johnstone R. W. (2014). New and Emerging HDAC Inhibitors for Cancer Treatment. J. Clin. Invest. 124 (1), 30–39. 10.1172/jci69738 24382387 PMC3871231

[B30] Xiao J. Gong Y. Chen Y. Yu D. Wang X. Zhang X. (2017). IL-6 Promotes Epithelial-To-Mesenchymal Transition of Human Peritoneal Mesothelial Cells Possibly through the JAK2/STAT3 Signaling Pathway. Am. J. Physiology-Renal Physiol. 313 (2), F310–f318. 10.1152/ajprenal.00428.2016 28490530

[B31] Yan B. Xie S. Liu Z. Luo Y. Zhou J. Li D. (2015). STAT3 Association with Microtubules and its Activation Are Independent of HDAC6 Activity. DNA Cel Biol. 34 (4), 290–295. 10.1089/dna.2014.2713 PMC4390050 25621430

[B32] Yang X. Tong Y. Yan H. Ni Z. Qian J. Fang W. (2018). High Intraperitoneal Interleukin-6 Levels Predict Peritonitis in Peritoneal Dialysis Patients: a Prospective Cohort Study. Am. J. Nephrol. 47 (5), 317–324. 10.1159/000489271 29779030

[B33] Yang X. Zhang H. Hang Y. Yan H. Lin A. Huang J. (2014). Intraperitoneal Interleukin-6 Levels Predict Peritoneal Solute Transport Rate: a Prospective Cohort Study. Am. J. Nephrol. 39 (6), 459–465. 10.1159/000362622 24854010

[B34] Zhang S. Huang Q. Cai X. Jiang S. Xu N. Zhou Q. (2018). Osthole Ameliorates Renal Fibrosis in Mice by Suppressing Fibroblast Activation and Epithelial-Mesenchymal Transition. Front. Physiol. 9, 1650. 10.3389/fphys.2018.01650 30524310 PMC6258720

[B35] Zhang T. Kee W. H. Seow K. T. Fung W. Cao X. (2000). The Coiled-Coil Domain of Stat3 Is Essential for its SH2 Domain-Mediated Receptor Binding and Subsequent Activation Induced by Epidermal Growth Factor and Interleukin-6. Mol. Cel Biol. 20 (19), 7132–7139. 10.1128/mcb.20.19.7132-7139.2000 PMC86266 10982829

[B36] Zhang Y. Kwon S. Yamaguchi T. Cubizolles F. Rousseaux S. Kneissel M. (2008). Mice Lacking Histone Deacetylase 6 Have Hyperacetylated Tubulin but Are Viable and Develop Normally. Mol. Cel Biol. 28 (5), 1688–1701. 10.1128/mcb.01154-06 PMC2258784 18180281

[B37] Zhou Q. Bajo M.-A. Del Peso G. Yu X. Selgas R. (2016a). Preventing Peritoneal Membrane Fibrosis in Peritoneal Dialysis Patients. Kidney Int. 90 (3), 515–524. 10.1016/j.kint.2016.03.040 27282936

[B38] Zhou X. Zang X. Ponnusamy M. Masucci M. V. Tolbert E. Gong R. (2016b). Enhancer of Zeste Homolog 2 Inhibition Attenuates Renal Fibrosis by Maintaining Smad7 and Phosphatase and Tensin Homolog Expression. Jasn 27 (7), 2092–2108. 10.1681/asn.2015040457 26701983 PMC4926973

[B39] Zhu S. Goldschmidt-Clermont P. J. Dong C. (2004). Transforming Growth Factor-β-Induced Inhibition of Myogenesis Is Mediated through Smad Pathway and Is Modulated by Microtubule Dynamic Stability. Circ. Res. 94 (5), 617–625. 10.1161/01.res.0000118599.25944.d5 14739161

