# Supplementary figures and images for: Requirement of Histone Deacetylase 6 for Interleukin-6 Induced Epithelial-Mesenchymal Transition, Proliferation, and Migration of Peritoneal Mesothelial Cells
Source: Front Pharmacol. 2021 Aug 30;12:722638. doi: 10.3389/fphar.2021.722638 (PMC8435636; doi:10.3389/fphar.2021.722638)

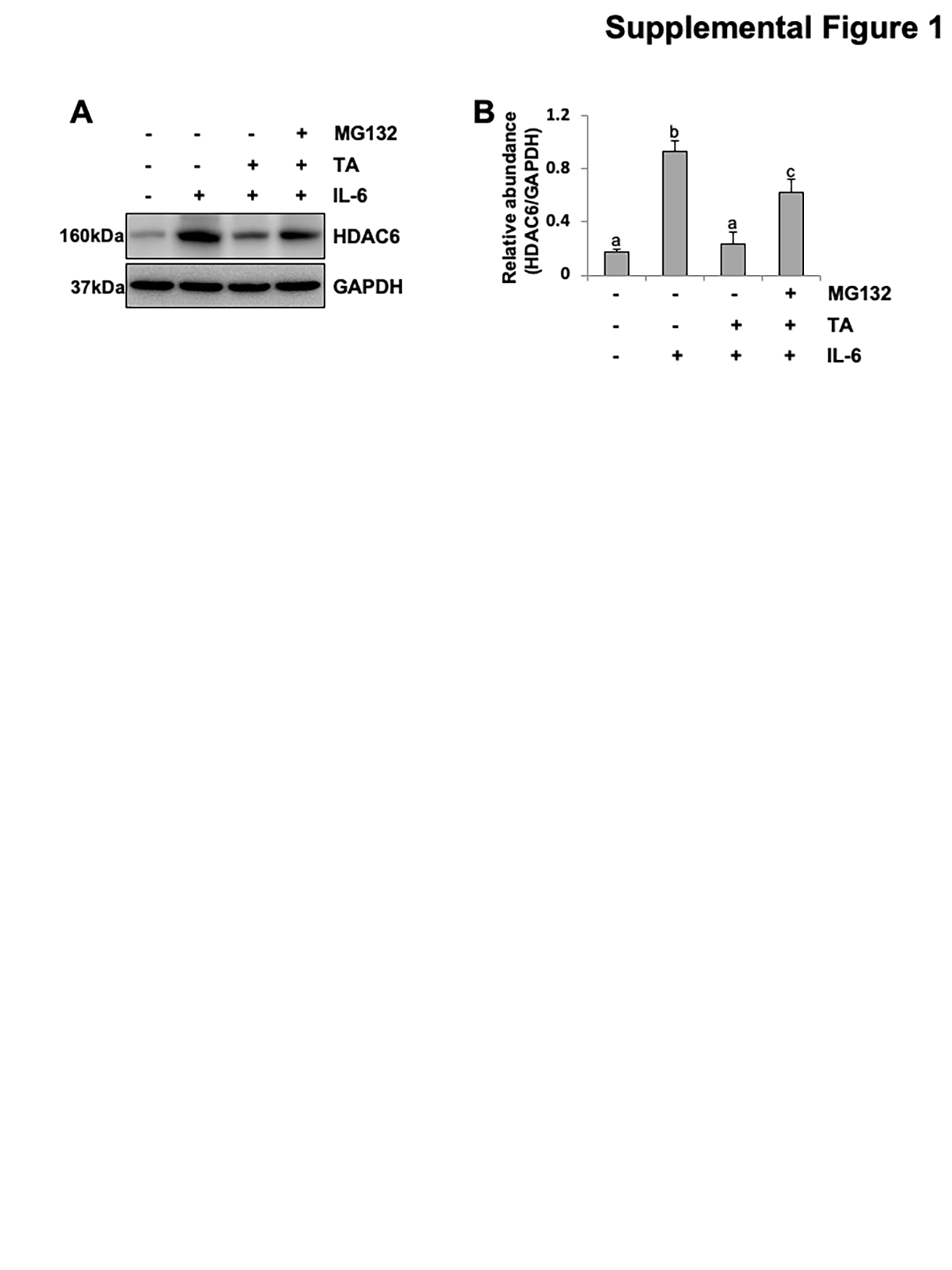

Supplement: Supplementary file 1 [file Image1.tiff]

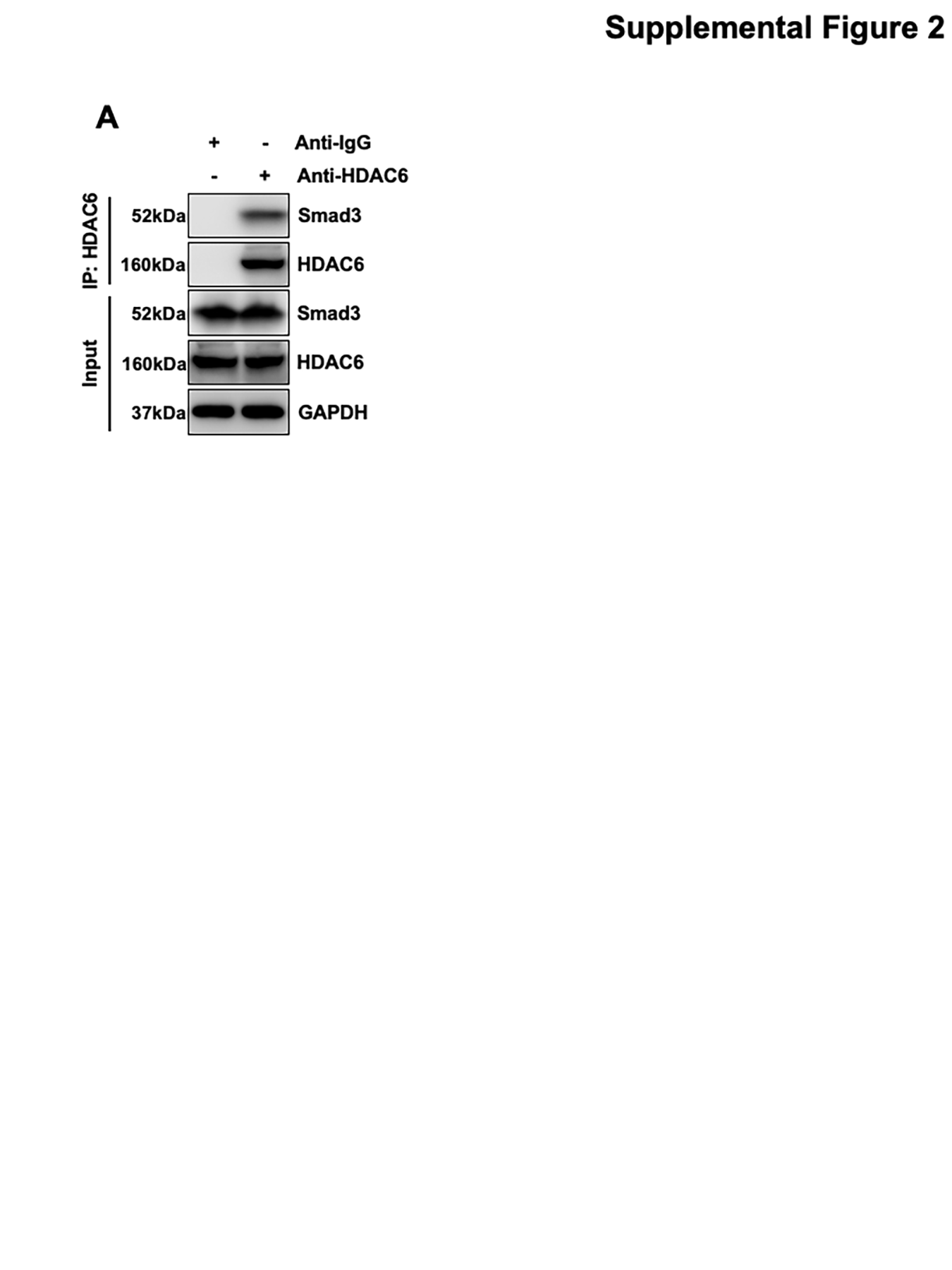

Supplement: Supplementary file 2 [file Image2.tiff]
